# Supplementary material for: Landscape of IGH germline genes of Chiroptera and the pattern of Rhinolophus affinis bat IGH CDR3 repertoire
Source: Microbiol Spectr. 2024 Mar 11;12(4):e03762-23. doi: 10.1128/spectrum.03762-23 (PMC10986613; doi:10.1128/spectrum.03762-23)
Supplement: Tables S1 to S5 and Figures S1 to S10 — Supplemental tables and figures. [file spectrum.03762-23-s0001.pdf]

Supplement Table S1 Determination of Cytb genotypes of Rhinolophus affinis.

Sample	Cytb Sequence	Identity	Score	Result
Bat1	AGATTCAAAGGAACGCATTTCGTCGATCTACCAGCCCCCTCAAGTATTTCCTCCTGATGAAACTTCG<br>GATCCCTCCTAGGGGTCTGCCTTGCTGTACAAATTATTACAGGCCTTTTCCTAGCTATACACTACACA<br>TCAGACACCGCCACAGCCTTCTACTCTGTAACCCATATCTGCCGAGACGTCAACTACGGCTGAGTCC<br>TACGCTACCTCCATGCCAACGGAGCCTCCATATTCTTTATCTGCCTGTTCCTACACGTAGGACGAGGG<br>ATCTACTATGGCTCCTATACATTTTCAGAAACATGAAACATCGGAATTATCCTCCTCTTCGCCGTCAT<br>AGCCACAGCATTTATGGGCTATGTACTTCCATGAGGCCAAATATCCTTCTGAGGGGCAACAGTCATT<br>ACAAACCTCCTCTCAGCCATCCCCTATGTAGGAACAACCCTAGTAGAATGAGTCTGAGGAGGATTCT<br>CAGTAGACAAGGCCACACTCACCCGATTCTTCGCCTGACTTCCTCCCA	99.57%	850	Rhinolophus affinis
Bat2	TAAACGGACGCATTCGTCGATCTACCAGCCCCCTCAAGTATTTCCTCCTGATGAAACTTCGGATCCCT<br>CCTGGGGGTCTGCCTTGCTGTACAAATTATTACAGGCCTTTTCCTAGCTATACACTACACATCAGACA<br>CCGCCACAGCCTTCTACCCCGTAACCCATATCTGCCGAGACGTTAACTACGGCTGAGTCCTACGCTA<br>CCTCCATGCCAACGGAGCCTCCATATTCTTTATCTGCCTGTTCCTACACGTAGGACGAGGGATCTACT<br>ATGGCTCCTATACATTTTCAGAAACATGAAACATCGGAATTATCCTCCTCTTCGCCGTCATAGCCACA<br>GCATTTATGGGCTATGTACTTCCATGAGGCCAAATATCCTTCTGAGGGGCAACAGTCATCACAAACC<br>TCCTCTCAGCCATCCCCTATGTAGGAACAACCCTAGTAGAATGGGTCTGAGGAGGATTCTCAGTAAA<br>CAAAGCCACACTCACCCGATTCTTCGCCTGACTTTCCTCCAA	99.10%	798	Rhinolophus affinis
Bat3	CTAGGTCATGACGCATTCGTCGATCTACCAGCCCCCTCAAGTATTTCCTCCTGATGAAACTTCGGATC<br>CCTCCTAGGGGTCTGCCTTGCTGTACAAATTATTACAGGCCTTTTCCTAGCTATACACTACACATCAG<br>ACACCGCCACAGCCTTCTACTCTGTAACCCATATCTGCCGAGACGTCAACTACGGCTGAGTCCTACG<br>CTACCTCCATGCCAACGGAGCCTCCATATTCTTTATCTGCCTGTTCCTACACGTAGGACGAGGAATCT<br>ACTATGGCTCCTATACATTTTCAGAAACATGAAACATCGGAATTATTCTCCTCTTCGCCGTCATAGCC<br>ACAGCATTTATGGGCTATGTACTTCCATGAGGCCAAATATCCTTCTGAGGGGCAACAGTCATCACAA<br>ACCTCCTCTCAGCCGTCCCCTATGTAGGAACAACCCTAGTAGAATGGGTCTGAGGAGGATTCTCAGT<br>AGACAAGGCCACACTCACCCGATTCTTCGCCTGACCTTCCTCCCA	99.38%	874	Rhinolophus affinis

Supplement Table S2 22 reverse IGHV genes in Phyllostomus discolor

Name	Minimum	Maximum	Length	Direction
IGHV(II)-2*02	2057623	2057921	299	Reverse
IGHV(III)-1	2661200	2661495	296	Reverse
IGHV(III)-2*01	2627687	2627979	293	Reverse
IGHV1-1	2671585	2671876	292	Reverse
IGHV1-2	2618909	2619204	296	Reverse
IGHV1-7	979077	979372	296	Reverse
IGHV3-1	2706384	2706682	299	Reverse
IGHV3-2	2674087	2674379	293	Reverse
IGHV3-3*01	2638006	2638301	296	Reverse
IGHV3-4	2513676	2513968	293	Reverse
IGHV3-5	2489863	2490155	293	Reverse
IGHV3-11*02	2025107	2025402	296	Reverse
IGHV3-13*01	2038667	2038964	298	Reverse
IGHV3-14	1961559	1961851	293	Reverse
IGHV4-1	2540331	2540629	299	Reverse
IGHV4-2	2533135	2533430	296	Reverse
IGHV4-3*04	2049536	2049831	296	Reverse
IGHV9-1	2579768	2580069	302	Reverse
IGHV9-3	965030	965332	303	Reverse
IGHV10-1*01	2600109	2600407	299	Reverse
IGHV11-1	2500176	2500468	293	Reverse
IGHV11-3	1348145	1348435	291	Reverse

Supplement Table S3 Statistics on the number, direction and Functionality of genes contained in the IGHV gene family of three species of bats.

IGHV subgroup	Rhinolophus ferrumequinum		Phyllostomus discolor		Pipistrellus pipistrellus	
	direction	total	direction	total	direction	total
IGHV1	+	14	+/-	15	+	4
IGHV2	+	6	+	0	+	0
IGHV3	+	13	+/-	24	+	25
IGHV4	+	5	+/-	10	+	7
IGHV5	+	0	+	0	+	0
IGHV6	+	0	+	0	+	0
IGHV7	+	0	+	0	+	3
IGHV8	+	0	+	0	+	0
IGHV9	+	0	+/-	9	+	4
IGHV10	+	0	+/-	4	+	8
IGHV11	+	2	+/-	9	+	0
IGHV(I)	+	0	+	0	+	0
IGHV(II)	+	0	+/-	5	+	3
IGHV(III)	+	1	+/-	5	+	3
Total	+	41	+/-	81	+	57
Functional	+	35	+/-	48	+	34
ORF	+	1	+	1	+	3
Pseudogene (P)	+	5	+/-	32	+	20
P/Proportion	+	12%	+/-	40%	+	35%

+ is forward,- is reverse, + /- exists in both forward and reverse directions.

Supplement Table S4 Functional determination of IGHV/IGHJ genes in three species of bats.

IGHV/ IGHJ	Pipistrellus pipistrellus			IGHV/IGHJ	Rhinolophus ferrumequinum			IGHV/IGHJ	Phyllostomus discolor		
	Functionality	Stop Codon	Defective RSS		Functionality	Stop Codon	Defective RSS		Functionality	Stop Codon	Defective RSS
IGHV1-2	P	●		IGHV1-3*03	P	●		IGHV1-1	P	●	
IGHV1-3	P	●		IGHV2-1*02	ORF		●	IGHV1-11	P	●	
IGHV1-4	P	●		IGHV3-1	P	●		IGHV1-14	P	●	●
IGHV3-3	ORF		●	IGHV3-5*01	P	●		IGHV3-3*02	P	●	
IGHV3-4	P	●	●	IGHV4-4	P	●		IGHV3-3*04	P	●	
IGHV3-14	P	●		IGHV(III)-1	P	●		IGHV3-16*01	P	●	
IGHV3-15	ORF		●					IGHV3-16*02	P	●	
IGHV3-17	P	●						IGHV3-17	ORF		●
IGHV3-22	P	●						IGHV4-1	P	●	
IGHV3-24	P	●						IGHV4-3*04	P	●	
IGHV3-25	ORF		●					IGHV4-3*05	P	●	
IGHV4-1	P	●	●					IGHV9-3	P	●	
IGHV4-2	P	●						IGHV9-4	P	●	
IGHV4-5	P	●						IGHV9-5	P	●	
IGHV7-1	P	●						IGHV9-6	P	●	●
IGHV9-1	P	●						IGHV9-7	P	●	
IGHV9-3	P	●						IGHV9-8	P	●	
IGHV(II)-1	P	●						IGHV9-9	P	●	●
IGHV(II)-2	P	●						IGHV10-2	P	●	

IGHV(II)-3	P	●		IGHV11-3	P	●	●
IGHV(III)-1	P	●		IGHV11-5	P	●	
IGHV(III)-2	P	●		IGHV11-6	P	●	●
IGHV(III)-3	P	●	●	IGHV11-9	P	●	
				IGHV(II)-1	P	●	
				IGHV(II)-2*01	P	●	
				IGHV(II)-2*02	P	●	
				IGHV(II)-3	P	●	●
				IGHV(II)-4	P	●	●
				IGHV(III)-1	P	●	
				IGHV(III)-2*01	P	●	
				IGHV(III)-2*02	P	●	
				IGHV(III)-3	P	●	●
				IGHV(III)-4	P	●	

Supplement Table S5 IGH CDR3 HTS data sheet of human and mouse.

Sample	IGH		Subclasses			
	Productive	Clonotype	Name	Productive	Clonotype	Clonotype/Productive
			IGG	322,460	36,705	11.38%
M1	2,724,205	374,307	IGA	639,297	71,378	11.17%
			IGM	1,762,448	266,224	15.11%
			IGG	368,040	52,336	14.22%
M2	3,133,607	543,367	IGA	725,174	103,608	14.29%
			IGM	2,040,393	387,423	18.99%
			IGG	192,903	20,497	10.63%
M3	1,528,623	194,248	IGA	324,177	38,988	12.03%
			IGM	1,011,543	134,763	13.32%
			IGG	23,626	2,560	10.84%
H1	413,204	28,955	IGA	218,596	9,793	4.48%
			IGM	170,982	16,601	9.71%
			IGG	17,625	2,205	12.51%
H2	329,736	26,111	IGA	207,771	8,683	4.18%
			IGM	104,340	15,223	14.59%
			IGG	19,984	2,524	12.63%
H3	275,755	22,595	IGA	154,748	8,635	5.58%
			IGM	101,023	11,437	11.32%

Supplement Figure S1 Comparative analysis of the results of IGG quality control primers and experimental primers in Rhinolophus affinis.

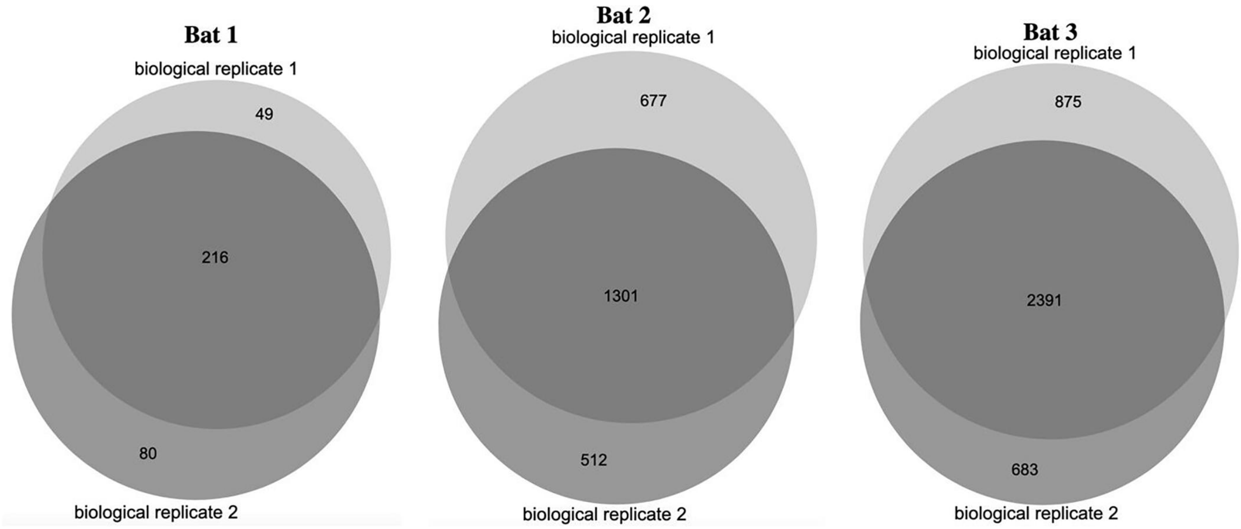

Supplement Figure S2 Amino acid sequence analysis of IGHV gene in three species of bats.

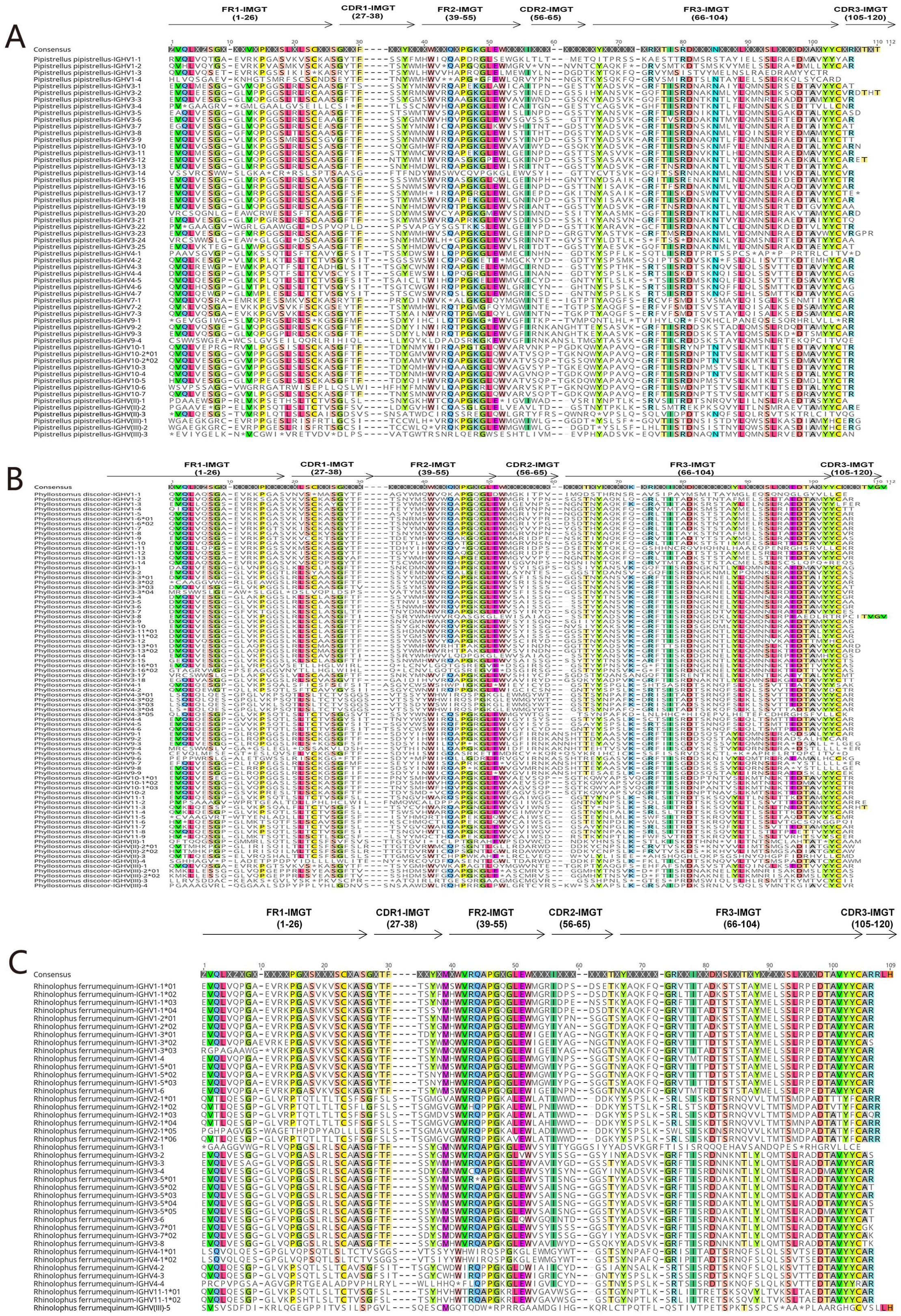

A: Pipistrellus pipistrellus; B: Phyllostomus discolor; C: Rhinolophus ferrumequinum.

Supplement Figure S3 Comparative analysis of IGHV genes in three bat species and other species.

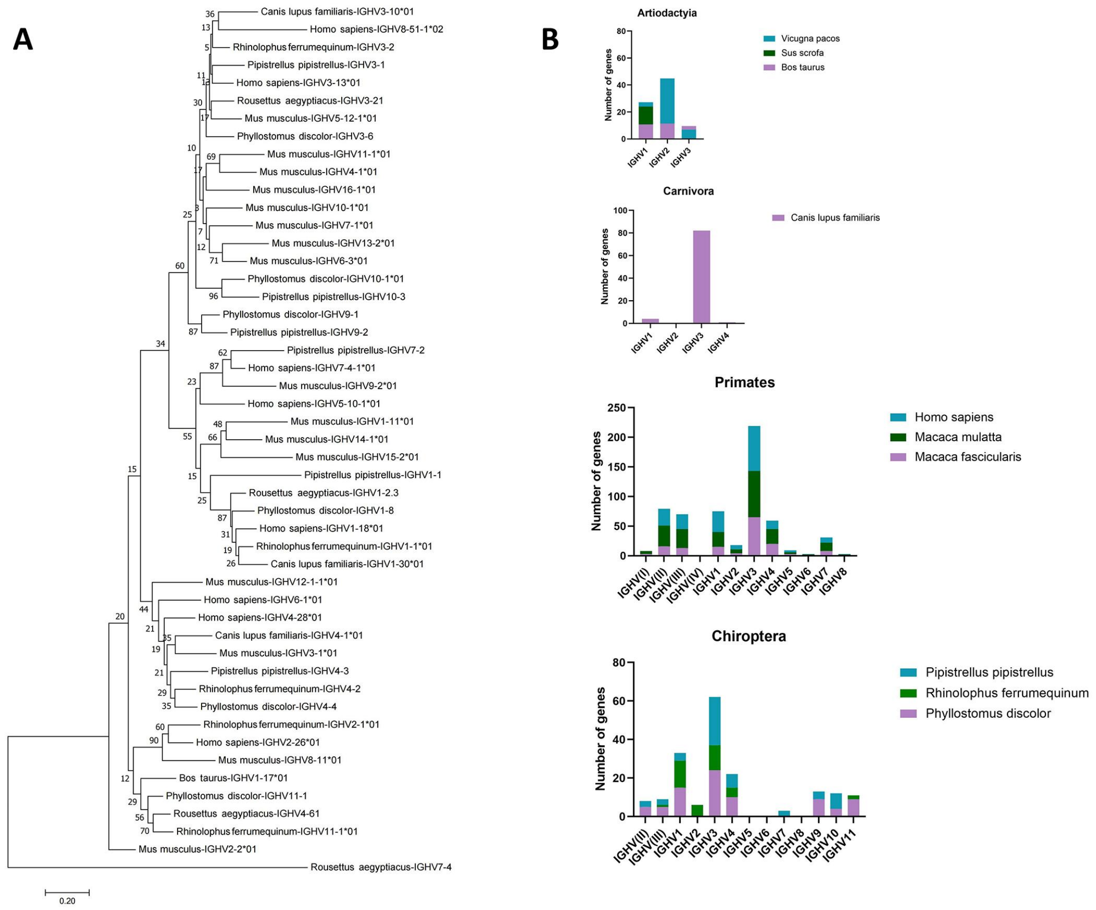

A: comparative analysis of IGHV gene phylogenetic tree of three species of bat, human, mouse, dog and cow. B: comparison of the number of IGHV gene families in bats, carnivores, primates and Artiodactyla.

Supplement Figure S4 IGH CDR3 HTS data Analysis of Network sharing Human and Mouse.

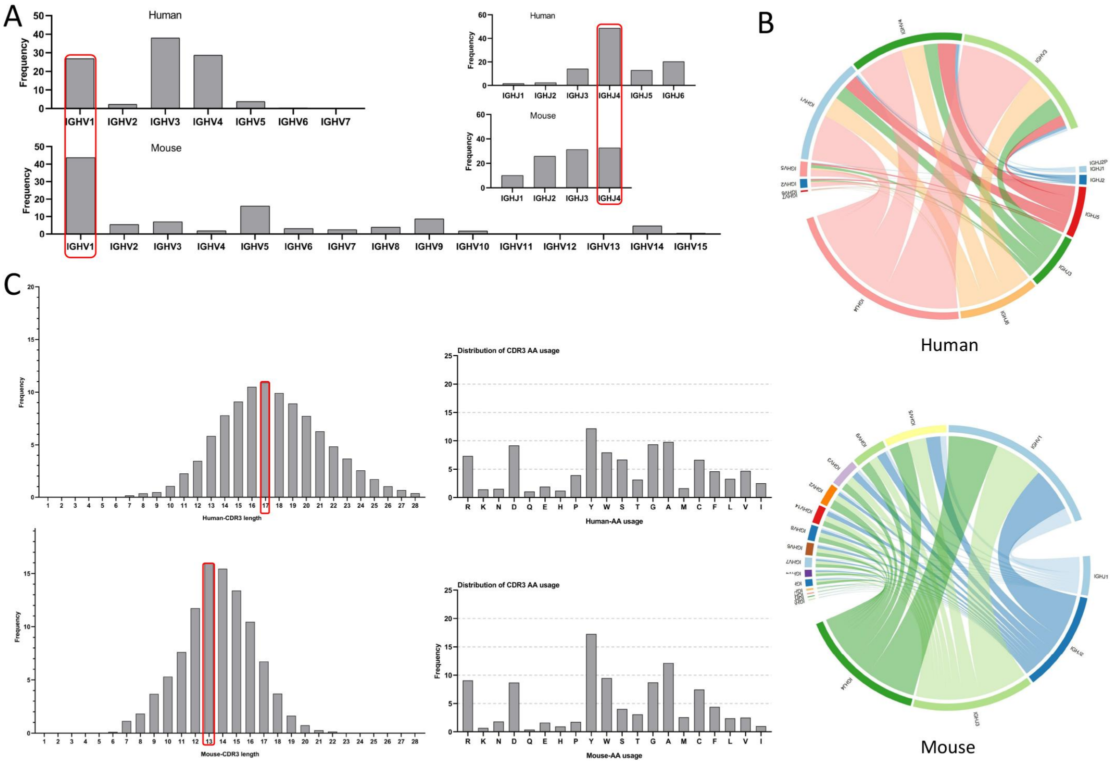

A: V and J usage analysis; B: V and J pairing analysis; C: Analysis of IGH CDR3 length and AA usage.

Supplement Figure S5 Analysis of V and J usage of Rhinolophus affinis, Human and Mouse IGM and IGE CDR3 Group of Rhinolophus affinis and Mouse.

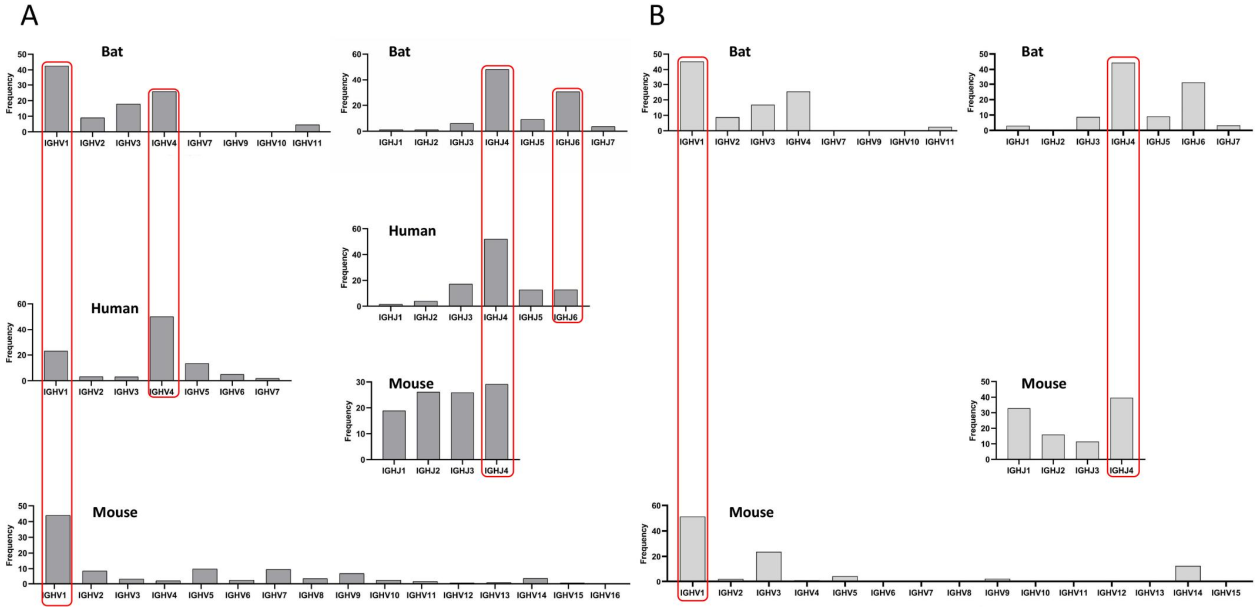

A: Analysis of V and J access of IGM CDR3 repertoire; B: Analysis of V and J access of IGE CDR3 repertoire.

Supplement Figure S6 V and J pairing of IGH CDR3 repertoire of Rhinolophus affinis, Human and Mouse.

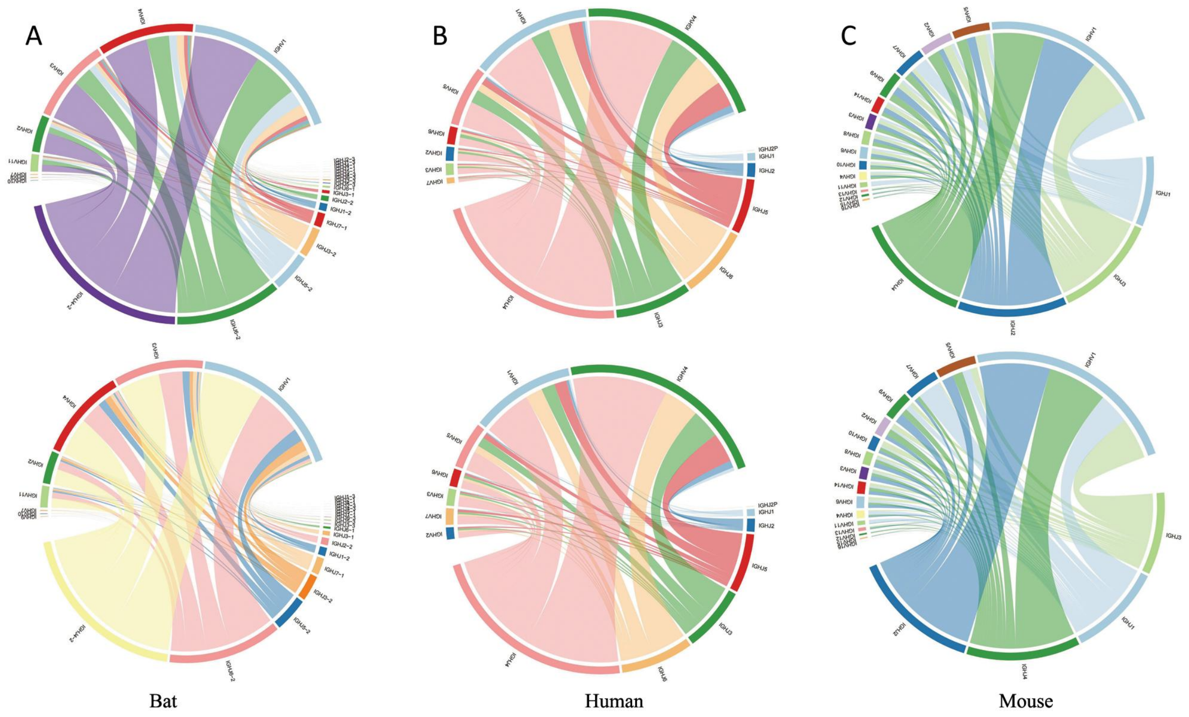

A: IGH CDR3 repertoire V and J pairing of B2 and B3; B: IGH CDR3 repertoire V and J pairing of H2 and H3; C: IGH CDR3 repertoire V and J pairing of M2 and M3.

Supplement Figure S7 Top clonal and relative abundance analysis, Overlap analysis of IGH CDR3 subspecies (IGA, IGM, IGG) in horseshoe bats, humans, and mice.

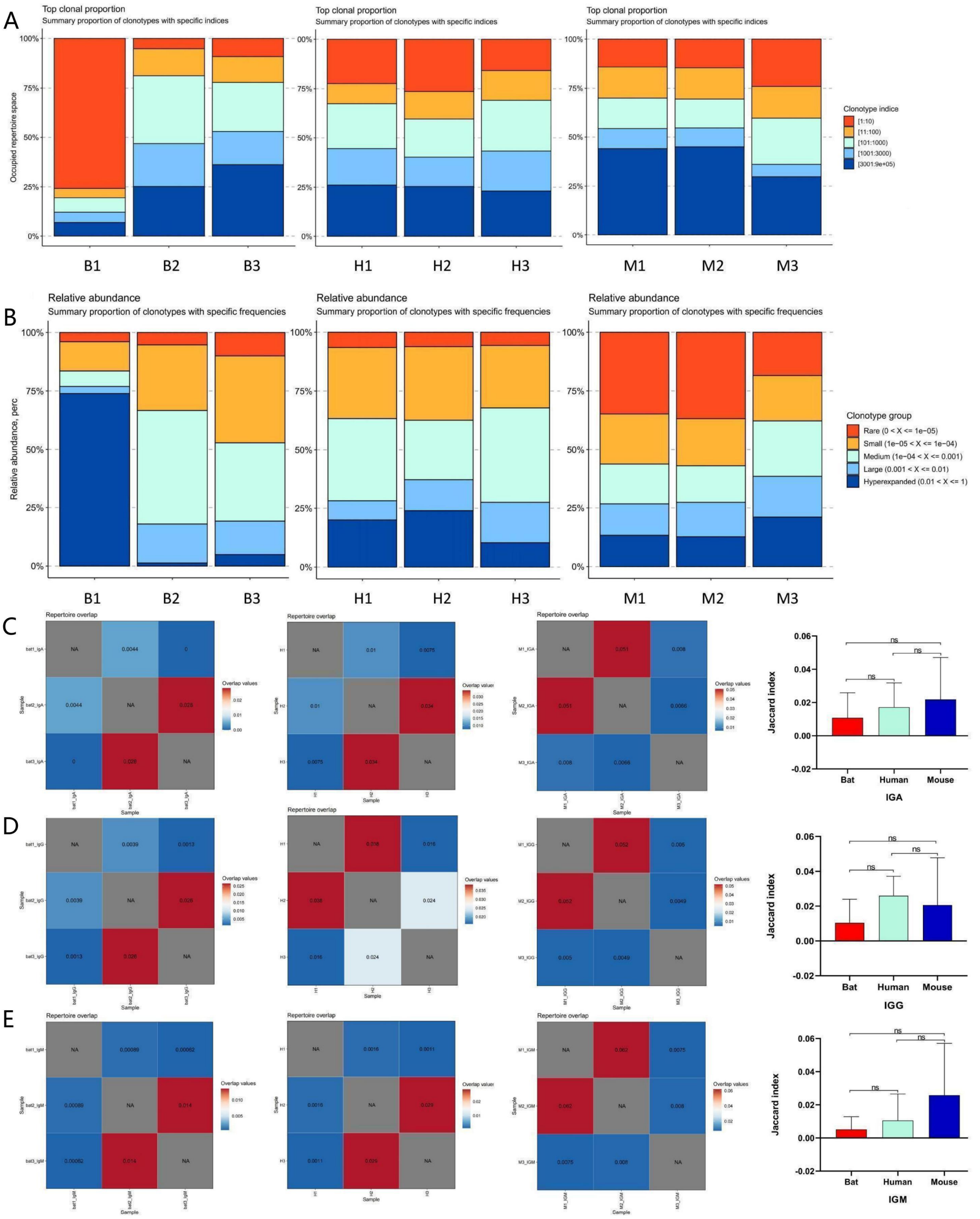

A: Top clonal analysis; B: Relative abundance analysis. C: Jaccard index of IGA; D: Jaccard index of IGG; E: Jaccard index of IGM. ns: P>0.05; *: P<0.05; **: P<0.01; ***: P<0.001.

Supplement Figure S8 Comparative Analysis of IGHCDR3 Total Motif among Rhinolophus affinis, Human and Mouse.(A:Bat,B:Human,C:Mouse)

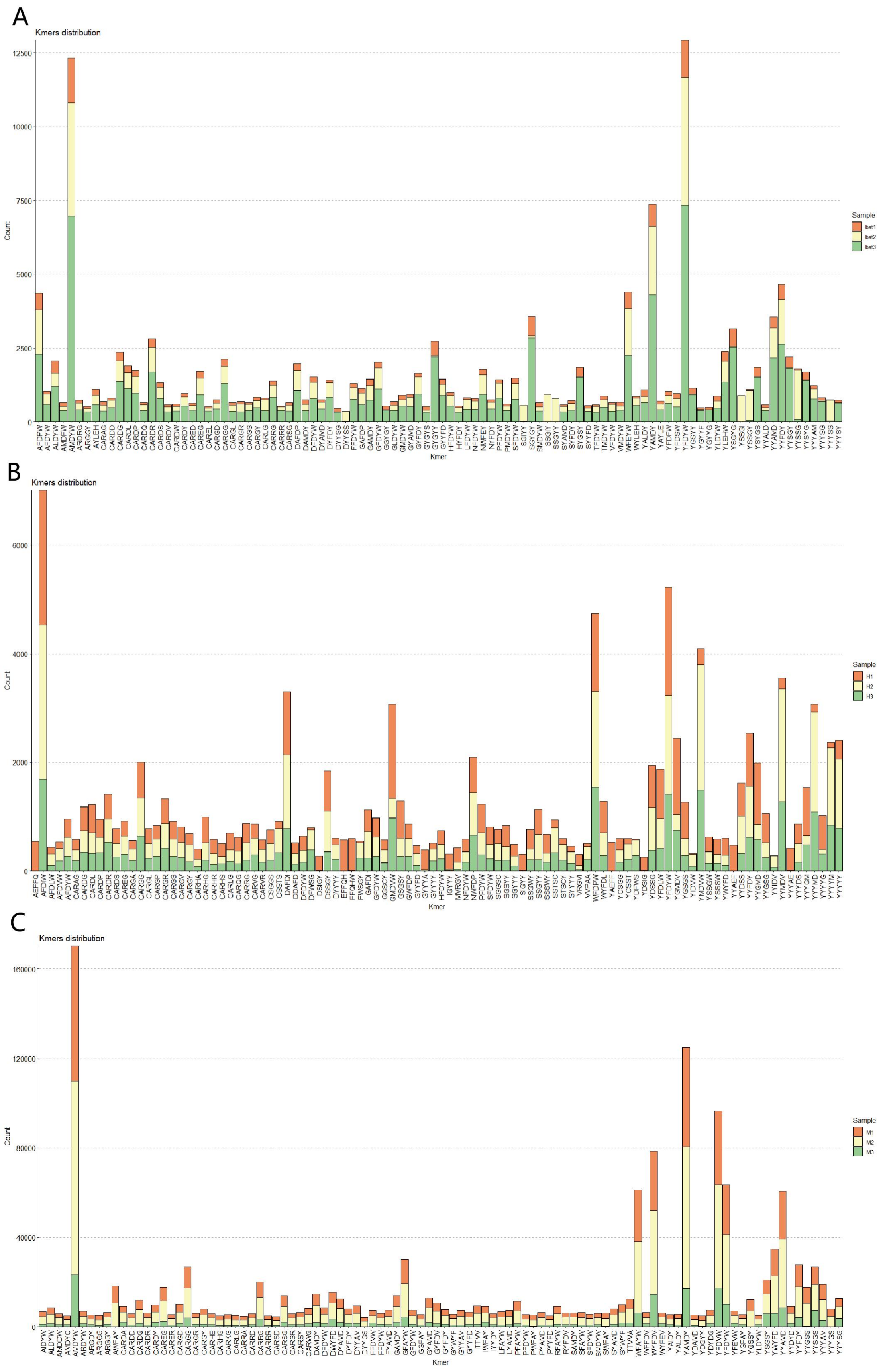

Supplement Figure S9 Bat, human, mouse IGH CDR3 subclass motif with the highest frequency of 10.(A:IGA,B:IGG,C:IGM)

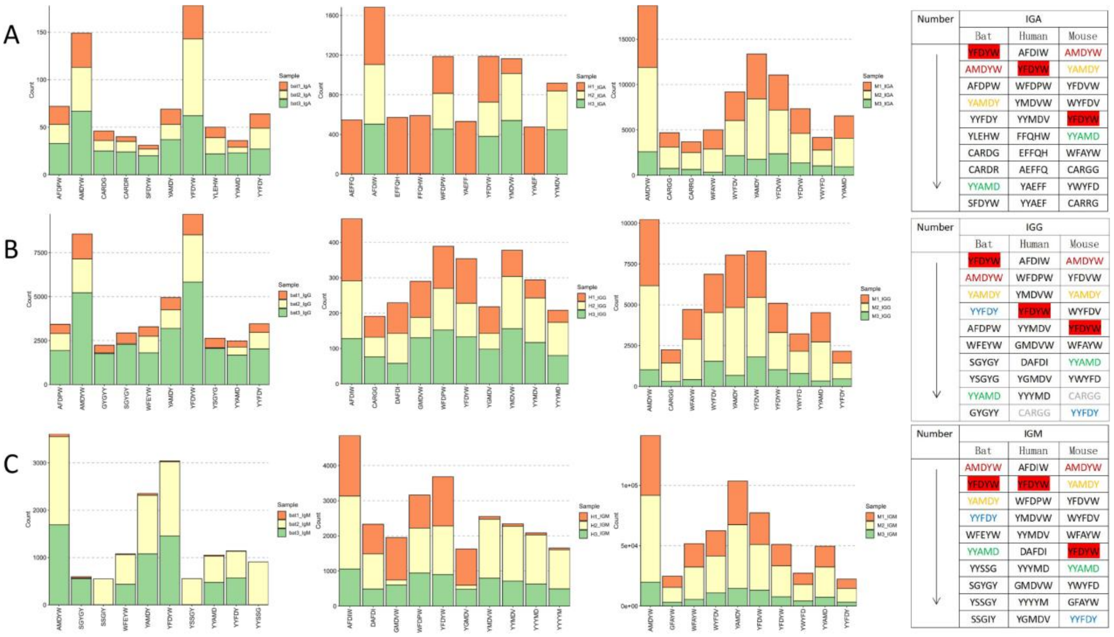

Number	IGA		
	Bat	Human	Mouse
↓	YFDYW	AFDIW	AMDYW
	AMDYW	YFDYW	YAMDY
	AFDPW	WFDPW	YFDVW
	YAMDY	YMDVW	WYFDV
	YYFDY	YYMDV	YFDYW
	YLEHW	FFQHW	YYAMD
	CARDG	EFFQH	WFAYW
	CARDR	AEFFQ	CARGG
	YYAMD	YAEFF	YWYFD
	SFDYW	YYAEF	CARRG

Number	IGG		
	Bat	Human	Mouse
↓	YFDYW	AFDIW	AMDYW
	AMDYW	WFDPW	YFDVW
	YAMDY	YMDVW	YAMDY
	YYFDY	YFDYW	WYFDV
	AFDPW	YYMDV	YFDYW
	WFEYW	GMDVW	WFAYW
	SGYGY	DAFDI	YYAMD
	YSGYG	YGMDV	YWYFD
	YYAMD	YYYMD	CARGG
	GYGYY	CARGG	YYFDY

Number	IGM		
	Bat	Human	Mouse
↓	AMDYW	AFDIW	AMDYW
	YFDYW	YFDYW	YAMDY
	YAMDY	WFDPW	YFDVW
	YYFDY	YMDVW	WYFDV
	WFEYW	YYMDV	WFAYW
	YYAMD	DAFDI	YFDYW
	YYSSG	YYYMD	YYAMD
	SGYGY	GMDVW	YWYFD
	YSSGY	YYYYM	GFAYW
	SSGIY	YGMDV	YYFDY

Supplement Figure S10 IGE overlap and motif Analysis of mice and bats.

A

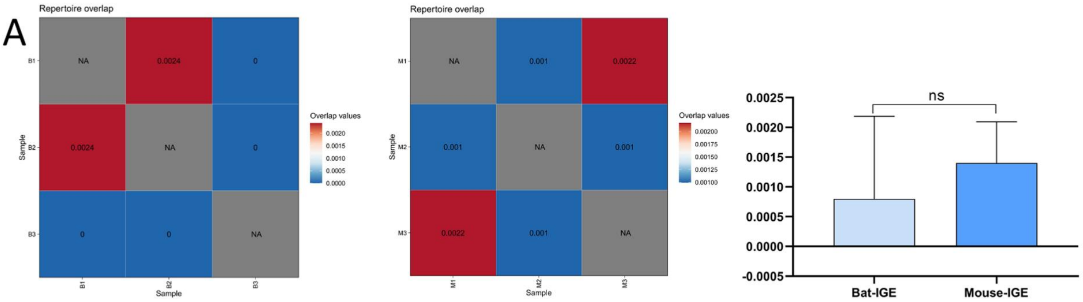

B

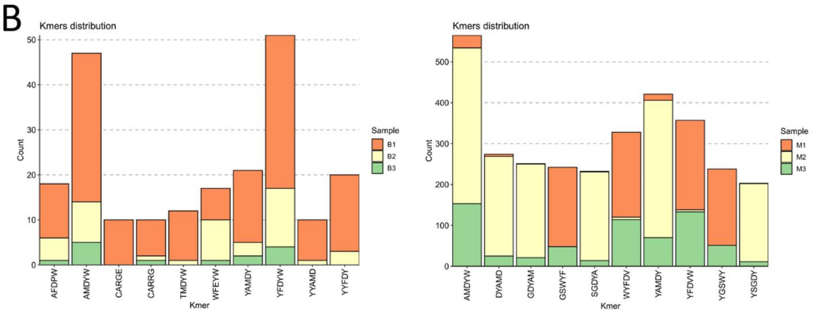

Number	IGE	
	Bat	Mouse
↓	YFDYW	AMDYW
	AMDYW	YAMDY
	YAMDY	YFDVW
	YYFDY	WYFDV
	AFDPW	DYAMD
	WFEYW	GDYAM
	TMDYW	GSWYF
	CARGE	YGSWY
	CARRG	SGDYA
	YYAMD	YSGDY

C

Number	BAT			
	IGA	IGG	IGM	IGE
↓	YFDYW	YFDYW	AMDYW	YFDYW
	AMDYW	AMDYW	YFDYW	AMDYW
	AFDPW	YAMDY	YAMDY	YAMDY
	YAMDY	YYFDY	YYFDY	YYFDY
	YYFDY	AFDPW	WFEYW	AFDPW
	YLEHW	WFEYW	YYAMD	WFEYW
	CARDG	SGYGY	YYSSG	TMDYW
	CARDR	YSGYG	SGYGY	CARGE
	YYAMD	YYAMD	YSSGY	CARRG
	SFDYW	GYGYY	SSGIY	YYAMD

A:IGE overlap Analysis of mice and bats. B:The 10 highest frequency motif in the IGE of mice and bats. C:The 10 motif with the highest frequency among the four subspecies of bats.
